# Cellulose Nanofibrils from Black Wattle (*Acacia mearnsii* De Wild.) Residues Produced by High-Intensity Ultrasonication: Production and Characterization

**DOI:** 10.1021/acsomega.5c10762

**Published:** 2026-02-02

**Authors:** Tereza Longaray Rodrigues, Paula da Cruz Pedroso, Marco Antônio da Fonseca Sobrinho, Wladimir Hernandez Flores, André Gündel, Ricardo Zottis, Luisa Bataglin Avila, Marcilio Machado Morais, Gabriela Silveira da Rosa, André Ricardo Felkl de Almeida

**Affiliations:** † School of Chemical Engineering, Laboratory of Biopolymers and Advanced Materials (BIOPOL), 28132 Universidade Estadual de Campinas (UNICAMP), Campinas, SP 13083-970, Brazil; ‡ Graduate Program in Materials Science and Engineering, Federal University of Pampa (UNIPAMPA), Bagé, RS 96413-172, Brazil; § Chemical Engineering, 186060 Federal University of Pampa (UNIPAMPA), Bagé, RS 96413-172, Brazil; ⊥ Graduate Program in Science and Engineering of Materials, Federal University of Pampa (UNIPAMPA), Bagé, RS 96413-172, Brazil

## Abstract

This study investigates the production of cellulose nanofibrils (CNF) from black wattle bark residues using high-intensity ultrasound (HIUS) as an environmentally friendly mechanical process. The residues were subjected to alkaline delignification and bleaching to obtain cellulose microfibers (CMF), which were subsequently ultrasonicated at different concentrations (1 and 2 wt %) and temperatures (4 and 25 °C). The resulting CNF suspension was characterized by atomic force microscopy (AFM), Fourier-transform infrared spectroscopy (FTIR), X-ray diffractometry (XRD), and thermogravimetric analysis (TGA). Only the 1 wt % CMF suspension treated at 25 °C exhibited effective fibrillation, yielding CNF with a diameter of 11.93 ± 4.83 nm. The CNF displayed characteristic functional groups, a crystallinity index of 52.21%, and thermal behavior consistent with that of lignocellulosic-derived nanomaterials. These findings confirm the suitability of black wattle bark residues as precursors for CNF production. Furthermore, utilizing these residues as a raw material represents a promising alternative to conventional cellulose sources, providing a more environmentally sustainable route for obtaining nanofibrils through an eco-friendly ultrasonication-based methodology.

## Graphical abstract

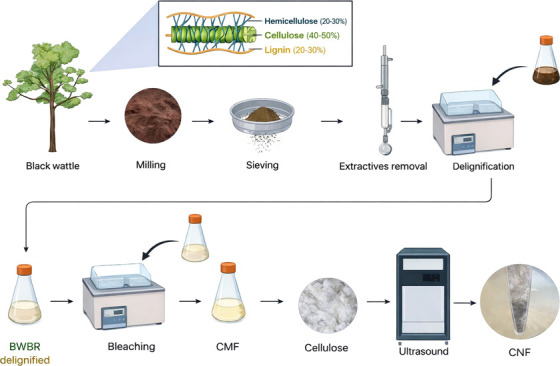

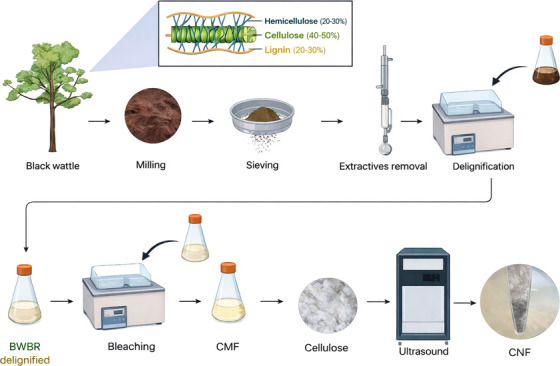

## 1 Introduction

The sustainable development goals (SDGs) have reinforced the need for environmentally responsible production routes, encouraging the development of eco-friendly materials and the valorization of renewable resources.^1–3^ In this context, the reuse of lignocellulosic waste has gained increasing attention as a strategy to convert underutilized biomass into high value-added materials,^4^ including adsorbents,^5–8^ food packaging,^9,10^ composites,^11,12^ building materials,^13,14^ and nanocellulose.^14,15^ Nanocellulose, composed of cellulose chains at the nanoscale, can be obtained as nanocrystals, nanofibrils, or bacterial nanocellulose, depending on the production route and resulting morphology.^16^ Several methodologies have been explored for its production, encompassing chemical, mechanical, and enzymatic processes, such as acid hydrolysis,^17^ microfluidization,^18^ high-intensity ultrasound,^19^ and enzymatic treatments.^20,21^

High-intensity ultrasound (HIUS) operates through a unique mechanism when compared with conventional mechanical fibrillation methods such as high-pressure homogenization, grinding, or refining.^24^ While traditional mechanical processes apply continuous and broadly distributed shear forces, HIUS generates highly localized and transient shear stresses arising from acoustic cavitation, a phenomenon characterized by the formation, growth and violent collapse of microbubbles that produce microjets, shock waves, and microturbulence within the liquid medium.^27^ These extremely intense and short-lived energy peaks induce the rupture of the fibrillar wall, the delamination of the cellulose lamellae, and the individualization of nanoscale structures with reduced diameters and increased surface area.^19^

In addition, unlike high-pressure homogenization, HIUS is not subject to clogging issues nor does it require multiple passes to achieve effective fibrillation. Its localized, pulsating mechanical action tends to produce shorter yet more homogeneous fibrils with improved colloidal dispersion.^24^ Taken together, these distinctive mechanical effects support the hypothesis that HIUS can promote more efficient fibrillation and achieve a higher degree of nanofibril individualization compared with conventional mechanical methods.^24–27^ Nanocellulose has versatile properties, such as biodegradability, high mechanical and thermal resistance, and elevated aspect ratio. These properties promote its application in several areas, for instance, 3D bioprinting,^28^ reinforcement of cementitious material,^29^ barrier multilayer film,^30^ hydrogel,^31,32^ adsorption,^33,34^ drug sensors,^35^ packaging films^36,37^ and nanocomposite.^38,39^

In the recent years, an increase on cellulose research was observed regarding the use of lignocellulosic sources as raw materials aiming to produce nanocellulose due to its availability as well as low cost,^16^ some examples are *Imperata brasiliensis* grass,^40^ sisal fibers,^41^ oat hull,^42^ rice straw,^24^ bamboo,^14^ sugar cane bagasse,^43,44^ pineapple crown,^45^ olive waste,^46^ wheat straw,^47,48^ hemp fibers,^49^ elephant grass,^50^ hop stem,^51^ ramie fibers,^52^ and black wattle bark.^53^ Black wattle (Acacia mearnsii De Wild.) is a native Australian species that has spread worldwide due to its high adaptability to different environmental conditions.^54,55^ In Brazil, its cultivation is predominantly concentrated in the state of Rio Grande do Sul, where in 2019 approximately 7.3% of forest plantations consisted of black wattle, totaling nearly 75,900 ha.^56^ Its commercial relevance is mainly associated with wood and bark production, the latter being widely exploited for tannin extraction owing to its high content of pure, high-quality condensed tannins.^55,56^

The tannin extraction industry generates large volumes of residues known as exhausted black wattle bark, which currently have no significant commercial application and may pose environmental concerns.^57,58^ According to the Brazilian Institute of Geography and Statistics, more than 200,000 tons of bark residues were produced in 2021 alone.^59^ Previous studies have investigated the reuse of this residue for the development of adsorbents,^57,58,60^ composites,^61–63^ phenolic compounds,^64–66^ fertilizers,^67^ corrosion inhibitors,^68^ cellulose,^69^ and cellulose nanocrystals.^53^

However, black wattle bark represents a technically challenging biomass for nanocellulose production due to its exceptionally high tannin content, complex polyphenolic matrix, and highly recalcitrant lignocellulosic structure.^54^ The bark contains a dense network of lignin and hemicellulose tightly bound to cellulose microfibers (CMF), forming a rigid and chemically resistant matrix that significantly limits cellulose accessibility.^56^ This structural entanglement, characterized by strong lignin–carbohydrate complexes (LCCs), makes delignification and purification steps more demanding, requiring more effective chemical or mechanical treatments to disrupt these interactions.^55^ Consequently, isolating celluloses suitable for fibrillation is considerably more difficult than in other lignocellulosic residues.^56^ The few existing studies on nanocellulose derived from this biomass report only the production of cellulose nanocrystals through acid hydrolysis, which partially overcomes these challenges but does not address fibrillation processes.^55^

Therefore, further research is required to evaluate the potential of this residue as a raw material for producing cellulose nanofibrils by using environmentally friendly methods. In this context, this study aims to produce cellulose nanofibrils (CNF) from black wattle bark residues through a green HIUS methodology and to characterize the resulting nanomaterials. The central hypothesis is that the HIUS process is capable of overcoming the structural recalcitrance of this tannin-rich biomass, enabling effective fibrillation and yielding CNF with morphological, structural, and thermal properties suitable for applications in advanced materials.

## 2 Materials and Methods

Black wattle (*Acacia mearnsii* De Wild.) bark residues (BWBR) used in this study were supplied by a tannin extraction industry located in southern Brazil. The chemicals used in this study were analytical grade: sodium hydroxide (Vetec), sodium chlorite (NaClO_2_, technical grade, 80%, Sigma-Aldrich), glacial acetic acid (Dinâmica), perchloric acid (Vetec), hydrogen peroxide (Dinâmica), potassium hydroxide (PQumicos), ethanol (Perfyl Tech), and nitric acid (Vetec).

### 2.1 Isolation of CMF from BWBR

Extraction of cellulose fibers from BWBR was carried out according to the procedure described by Rodrigues et al.^69^ BWBR were milled to sieve aiming to obtain a fraction composed by particles <0.25 mm, then it was dewaxed by sequential extraction with hexane/ethanol/water in Soxhlet for 6 h followed by drying at oven for 18 h at 50 °C. The delignification step was performed by the treatment of dewaxed BWBR with 6% wt. NaOH solution at a liquid to solid ratio of 33.33 mL/g at 65 °C for 2.5 h. Then, it was vacuum filtered, neutralized, and dried at 50 °C for 18 h. Subsequently, the delignified sample was bleached with a 1.7 wt % sodium chlorite solution and acetate buffer, using a liquid-to-solid ratio of 100 mL/g, at 80 °C for 4 h. Afterward, the sample was vacuum filtered, neutralized, and dried at 50 °C for 18 h, obtaining the CMF from BWBR, as illustrated by Figure 1.

**Figure 1:**
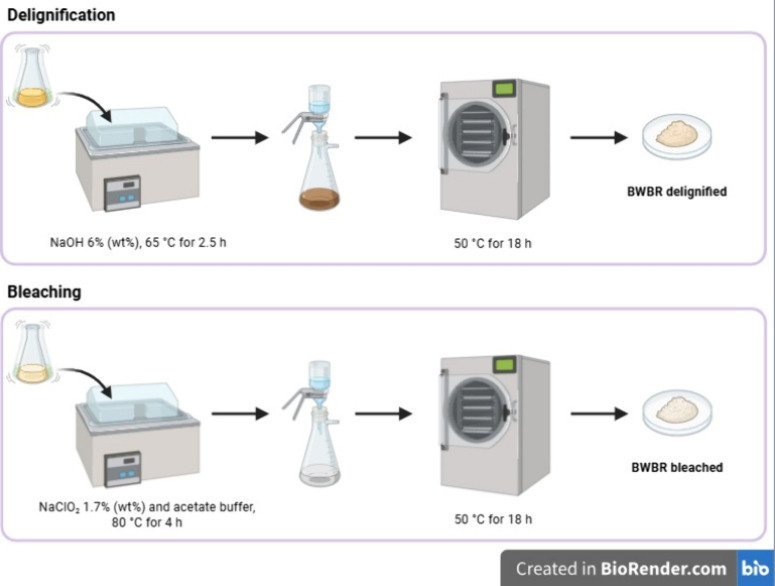
Production of CMF.

### 2.2 Production of CNF

The CMFs were soaked in 50 mL of distilled water with fiber concentrations of 1 and 2% (wt %) for 24 h and then treated by HIUS (ECOSONICS—ULTRASONIC QR 500, Brazil). The experimental conditions involved the use of a 20 mm diameter probe operating at 20 kHz and 550 W in continuous mode for 30 min at 90% amplitude to produce CNF. The probe was positioned 0.5 cm above the internal bottom surface of the beaker throughout the entire process. Active cooling was applied during sonication using a metabolic bath, as summarized in Table 1. Subsequently, the samples were centrifuged at 3000 rpm for 10 min to separate the nanofibers (top layer) from the microfibers (bottom layer), as illustrated in Figure 2. The CNF were collected and stored at 4 °C until characterization by atomic force microscopy (AFM), according to the procedure described by Cheng et al.^23^

**Figure 2:**
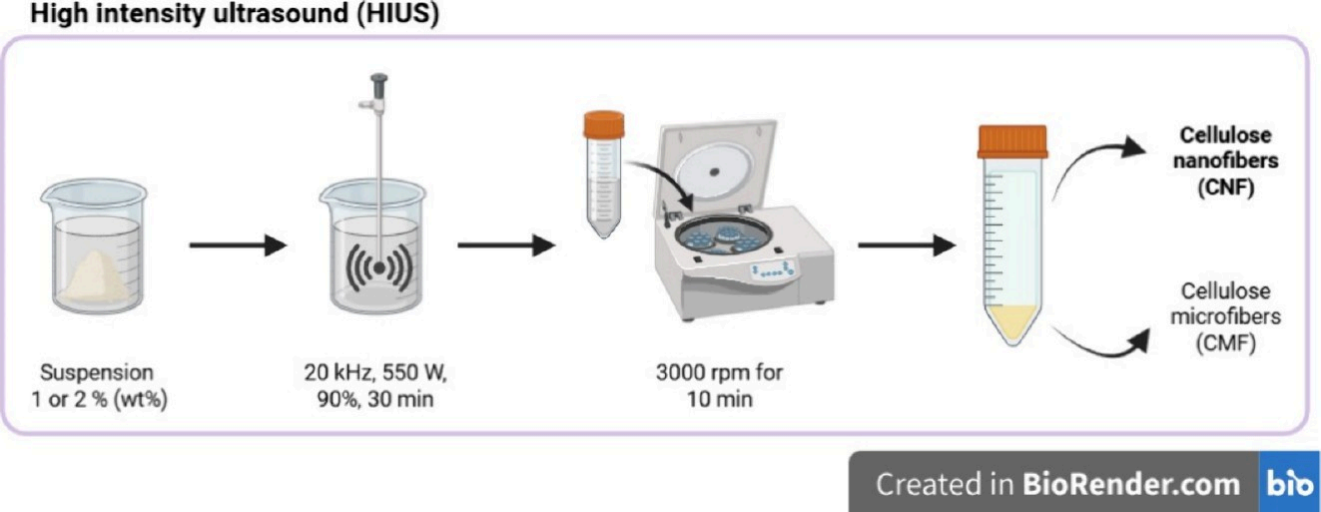
Production of cellulose nanofibers by HIUS.

**Table 1:** Experimental Conditions for CNF Production

	concentration (wt %)	temperature (°C)
CNF1	1	25 ± 2
CNF2	1	4 ± 2
CNF3	2	25 ± 2
CNF4	2	4 ± 2

To ensure consistency and reproducibility in the calculation of mass and yield, all samples were handled on a dry-mass basis (*Y*). The CMF obtained after the bleaching stage was oven-dried at 50 °C for 18 h (*m*_CMF_), and its dry mass was recorded to establish the initial solids content. After the HIUS treatment and the subsequent centrifugation step, the nanofibril-rich supernatant was separated, frozen, and freeze-dried to obtain the dry mass of CNF (*m*_CNF_). Moisture correction was therefore inherent to the process, since both CMF (before HIUS) and CNF (after HIUS) were weighed in their dried state, ensuring accurate determination of solids content.

The yield was calculated with respect to the post-HIUS fraction, considering the mass of dried CNF relative to the initial mass of CMF used in each experiment. All analyses were performed using three independent replicates per condition, and the reported yield values represent the mean ± the standard deviation.

$$Y(\%)=\frac{m_{\mathrm{CNF}}}{m_{\mathrm{CMF}}}\times 100$$ 1

### 2.3 CMF and CNF Characterization

Morphological analysis was evaluated by AFM, using the Agilent Technologies 5500 equipment at room temperature, by contact mode, with tips PPP-CONT (NANOSENSORS, force constant 0.2 N/m, resonance frequency 13 kHz). Images were captured using PicoView 1.14.4 software (MOLECULAR IMAGING CORPORATION), and the nanoparticles’ dimensions were investigated using Gwyddion software, collecting 65 points. The preparation of samples was carried out by the drop of 1 μL of a diluted CNF suspension at a mica surface that was dried at room temperature.

Aiming to quantify the crystallinity index (CI), the samples were analyzed by an X-ray diffractometer (Rigaku, Model ULTIMA IV, Japan) based on the diffracted intensity data, as indicated by Segal et al.^70^ The *CrI* was quantified as indicated by eq 2, where *I*_002_ is the crystalline portion at a diffraction angle of 2θ ≈ 22.5°, and *I*_am_ is related to the intensity of the amorphous phase located at the lowest intensity of a diffraction angle at 2θ ≈ 18°.

$$\mathrm{Cr}\;I(\%)=\frac{I_{002}-I_{\mathrm{am}}}{I_{002}}\times 100$$ 2

Thermal stability of samples was evaluated by a thermogravimetric instrument (Shimadzu, TGA 50, Kyoto, Japan) considering the following conditions: approximately 5 mg of sample were heated in a platinum pan at 30–650 °C with a heating rate of 15 °C.min^–1^, and a nitrogen flow of 50 mL min^–1^. The isotherms employed were 100 °C for 30 min and 650 °C for 30 min.

Regarding the determination of functional groups, it was investigated through Fourier-transform infrared (FTIR) spectroscopy (IR-Prestige, Shimadzu, Japan) coupled with a diffusive reflection accessory. A KBr pastille was analyzed considering a resolution of 4 cm^–1^, from 400 to 4000 cm^–1^.

## 3 Results and Discussion

Figure 3 summarizes the morphologic aspect of each sample, which indicated the production of nanocellulose only under the CNF1 conditions due to the absence of nanoparticles under other experimental conditions. The results indicated a clear influence of temperature on nanoparticle production, where the increase in temperature from 4 °C (CNF2) to 25 °C (CNF1) enhanced cellulose fibrillation, resulting in the production of cellulose particles in the nanometric scale. This behavior is in accordance with that previously reported in the literature.^71^

**Figure 3:**
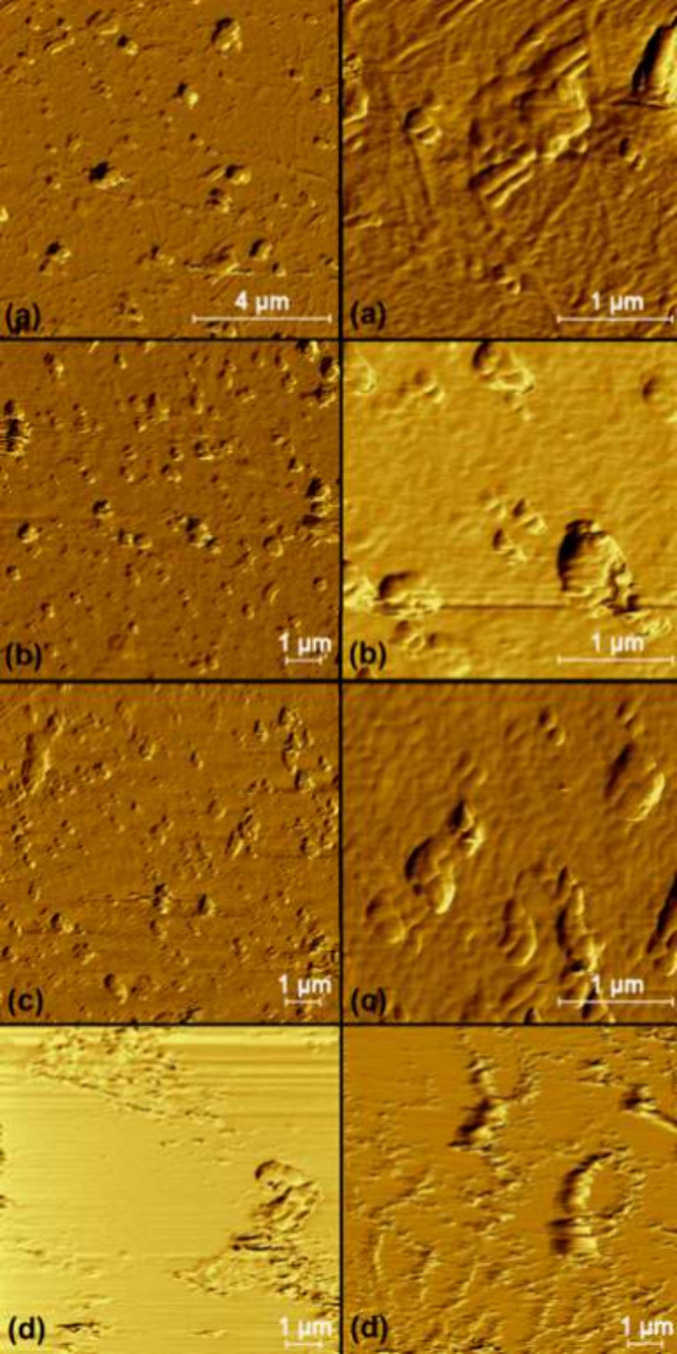
Morphological aspect of (a) CNF1, (b) CNF2, (c) CNF3, and (d) CNF4.

Regarding the influence of the suspension concentration, it was possible to observe that the increase in this factor negatively affected the cellulose fibrillation. This behavior may be related to the fact that when the CMF concentration increases, the aquatic force generated by the microbubbles cannot promote the agitation of cellulose fibers present in suspension, resulting in the low contact of some fibers with the probe tip.^71^ Furthermore, the presence of small, globular-shaped particles in all samples and this phenomenon can be attributed to two plausible hypotheses. The first one considers that these particles consist of microcrystalline cellulose, which were not affected by the ultrasound procedure and remain in suspension even after the centrifugation step. The other hypothesis suggests that the presence of lignin nanoparticles, which remained from biomass pretreatment and was also fibrillated by HIUS, promoting the production of nanolignin, as also noticed by Yang et al.^47^ and Ewulonu et al.^21^ The second hypothesis is in agreement with the thermogravimetric analysis (TGA) (Figure 5c,d), which confirmed the presence of this compound on raw material used for CNF production by the presence of a mass loss near 450 °C, and was also observed on previous research,^69^ this aspect will be discussed in the following sections.

The value observed in this work was comparable to that reported by Wang et al.^71^ for the production of cellulose nanofibers from lyocell via HIUS. Nevertheless, the production of CNF from black wattle bark by HIUS yielded 3.01 ± 0.53%, a value substantially lower than most studies listed in Table 2, in which yields range from 10.8% (orange bagasse) to 89.35% (ramie fiber).

**Table 2:** Yield, Dimensions, and Crystallinity Index (CrI) of CNF Produced by HIUS Method

raw material	conditions	yield (%)	diameter (nm)	CrI_CMF_ (%)	CrI_CNF_ (%)	reference
black wattle bark	1 wt %%, 20 kHz, 550 W, 90%, 30 min	3.01 ± 0.53	9–28	88.14	52.21	this work
oat hull	10 wt %%, 20 kHz, 50%, 1.27 cm, 15 min	60–65				^42^
ramie fiber	2 wt %%, 22 kHz, 400 W, 1.5 cm, 60 min	83.90–89.35		62.50	73.65	^52^
canola straw fibers	1 wt %%, 20 kHz, 1200 W, 20 mm	36.45–46.07				^72^
orange bagasse	20 kHz, 750 W, 20%, 15 min	10.8				^73^
lyocell	1 wt %%, 1500 W, 30 min	3–5				^71^
sugar cane bagasse	0.5% (wt %), 1–3 h in H_2_O_2_, 20 kHz, 13 mm, 750 W, 70%		6–100	45.4	52.7–61.6	^44^
bamboo	0.25–0.5% (wt %), 19.5–20.5 kHz, 30%, 10–80 min		37–133			^75^

Several factors quantitatively contribute to this difference. First, the initial fiber size used in this work tends to be larger due to the lignocellulosic nature of black wattle bark and the presence of detectable lignin residues, as confirmed by FTIR and TGA. Studies employing purer or more refined fibers, such as ramie^52^ (83.9–89.35%) and canola straw fibers^72^ (36.45–46.07%), use materials with lower lignin content, which facilitates fibrillation. Second, the effective power applied in the present study (550 W at 20 kHz) is lower than that used in studies reporting higher yields, such as lyocell^71^ (1500 W; 3–5%) and canola straw fibers (1200 W; 36–46%). Although the yield obtained from lyocell is also low, the material has high purity and minimal lignin, indicating that in the case of black wattle bark, the limiting factor is not solely the applied power but rather the combination of power and the structural composition of the fiber. Another key factor is the specific cavitation energy, which depends on the interplay among power, probe diameter, suspension concentration, and viscosity. In the present work, a 1% (wt %) suspension was used, whereas Debiagi et al.,^42^ when producing CNF from oat hull at 2% (wt %), obtained substantially higher yields (60–65%). This demonstrates that higher concentrations enhance the mechanical energy transfer during fibrillation. Furthermore, the residual lignin content of black wattle bark confirmed by the presence of lignin nanoparticles in AFM images and by the third degradation stage in the TGA reduces fibrillation efficiency, as lignin increases structural rigidity and hinders the uniform propagation of cavitation waves.

The HIUS promoted the production of cellulose nanoparticles with diameter of 11.93 ± 4.83 nm (Figure 4), which were classified as CNF due to their morphological characteristics^16^ and are following other authors, as illustrated in Table 2.

**Figure 4:**
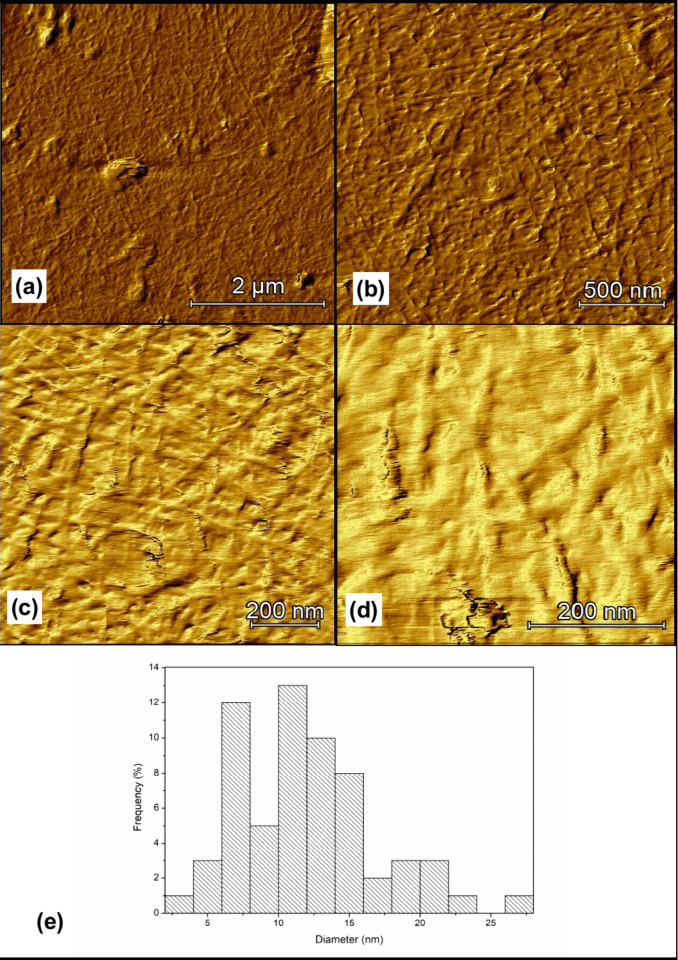
Morphological aspect of CNF: (a) 5 × 5 μm, (b) 2 × 2 μm, (c) 1 × 1 μm, and (d) 0.5 × 0.5 μm and (e) diameter distribution of CNF.

The difference between the values observed in the present work and those reported by the literature may be related to the raw material as well as to the experimental conditions of HIUS stage, where the employment of higher experimental time and intensity is related to a decrease on nanoparticles dimension due to the cavitation mechanism.^47,51,71^ The intertwined fiber structure was also observed by other authors^44,49,74^ and may be related to an increase in the superficial area of fibers, which promoted a strengthening of intermolecular hydrogen bonds, as well as the hydrophilic interaction between cellulose molecules.^44,75^

Figure 5a illustrates the X-ray diffractogram pattern, where the peaks located near 2θ ≈ 15°, 16.5°, 22.5°, and 34.3° may be attributed to crystalline planes, (1–10), (110), (102), (200), and (004), respectively, which are commonly observed on cellulose from native sources.^79,80^ It was possible to observe that these peaks were maintained after the ultrasonication step, indicating that the procedure did not affect the cellulose polymorph.^51^ Regarding peaks around 2θ ≈ 24°, 30°, and 38°, they can be related to the presence of quartz.^81^ The crystallinity index of CMF was equal to 88.14%, which is per the value observed in previous research with the same material.^69^ It was noticed that the HIUS step promoted an intensity decrease of the peaks, which characterizes the crystalline planes of cellulose. Szymańska-Chargot et al. (2022) observed that applying HIUS to cellulose extracted from hop stems reduced its crystallinity from 67% to approximately 60%, indicating that ultrasonic cavitation disrupts crystalline regions and increases the amorphous fraction. Similarly, in the study by Zhang et al. (2022), the applied treatment resulted in broadened diffraction peaks and a decrease in cellulose crystallinity, indicating the disruption of crystalline domains and partial amorphization of the material. The authors emphasize that these structural modifications are typical of processes involving extensive intermolecular bond cleavage and microfibril reorganization. This behavior resulted in an alteration on crystallinity index to 52.21% and was also reported by other authors^51,82,83^ Szymańska-Chargot^51^ observed that applying HIUS to cellulose extracted from hop stems reduced its crystallinity from 67% to approximately 60%, indicating that ultrasonic cavitation disrupts crystalline regions and increases the amorphous fraction. Similarly, in the study by Perdoch et al.,^83^ the applied treatment resulted in broadened diffraction peaks and a decrease in cellulose crystallinity, indicating the disruption of crystalline domains and partial amorphization of the material. The authors emphasize that these structural modifications are typical of processes involving extensive intermolecular bond cleavage and microfibril reorganization.

**Figure 5:**
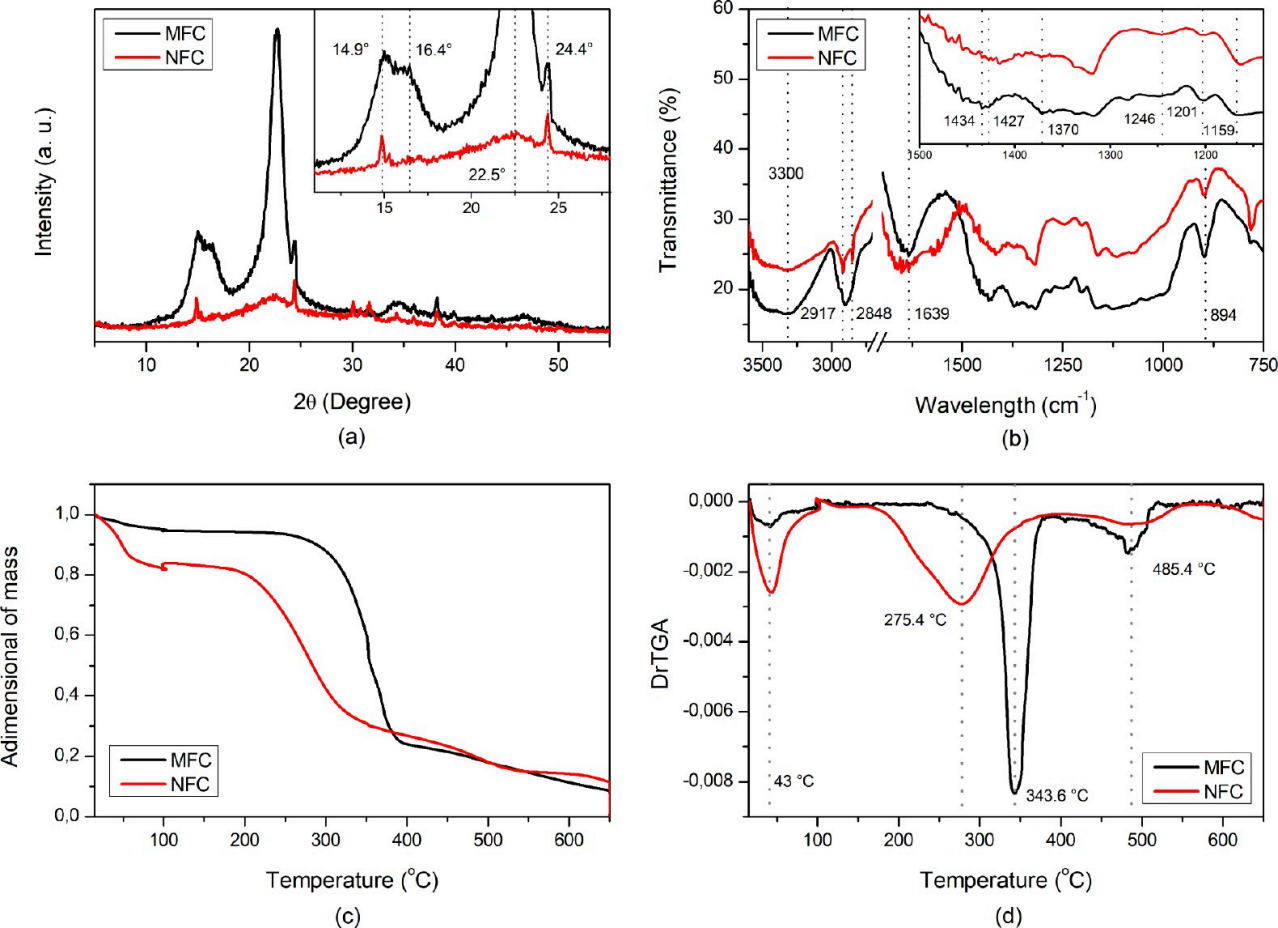
Characterization of CMF and CNF by (a) X-ray diffractometry, (b) FTIR spectroscopy, (c) TGA, and (d) derivative curves of TGA.

Szymanska-Chargot et al.,^51^ for example, reported a reduction in this parameter from 67 to 58.7% during the production of CNF from hop cones via HIUS. This behavior may be related to the mechanism that conducts the HIUS, which involves the production of bubbles responsible for the disruption of cellulose microfibers to generate the nanofibrils.^74^ According to Li et al.,^82^ HIUS is considered a nonselective procedure that promotes the removal of both amorphous and crystalline phases of cellulose, where the decrease on index crystallinity may be attributed to break of crystallites that composes the crystalline phase of cellulose.^51^ A hypothesis for this decrease may be related to the raw material crystallinity index. The material evaluated as raw material in the present work has a high crystallinity index; since CMF was composed of a rich-phase crystalline cellulose, it had a small fraction of amorphous cellulose to be degraded by HIUS, which, associated with its ability to degrade even crystalline phases, resulted in the reduction on crystallinity index.

The implications of this reduced crystallinity index are relevant for the structural and functional behavior of the produced CNF.^52^ A lower crystallinity fraction generally decreases the stiffness and elastic modulus of cellulose, as the crystalline domains are chiefly responsible for mechanical rigidity. Conversely, the relative increase in amorphous content enhances flexibility and may favor applications requiring deformability, such as hydrogels, flexible films, and polymeric matrices.^51^ Moreover, amorphous regions exhibit higher chemical accessibility, which increases the reactivity of the CNF toward functionalization, surface modification, and interactions in composite systems.^50^ The lower crystallinity can also improve water dispersibility and interfacial affinity with hydrophilic matrices, potentially broadening applications in coatings, biodegradable packaging, and adsorption-based systems. Thus, although the reduction in the crystallinity index may limit applications requiring maximum mechanical reinforcement, it can be advantageous for technologies that rely on higher reactivity, structural adaptability, or enhanced interaction with surrounding media.

FTIR analysis of CMF and CNF samples (Figure 5b) revealed only a few structural changes that are directly associated with the ultrasonic treatment. The broad band near 3300 cm^–1^, assigned to O–H stretching, showed a slight decrease in intensity after HIUS, suggesting modifications in hydrogen-bonding interactions on the fiber surface.^42,49–51,84^ A reduction in the band at 1430 cm^–1^, attributed to CH_2_ deformation related to cellulose crystallinity, indicates partial disruption of ordered domains induced by the ultrasonic process.^51^ The band at approximately 1370 cm^–1^, corresponding to C–H deformation, also decreased in intensity, which is consistent with fibril size reduction and increased exposure of surface groups.^50,51,84^ Overall, these spectral changes confirm that HIUS altered the chemical and supramolecular environment of cellulose, facilitating fibrillation and the formation of nanofibers, while preserving the fundamental functional groups of the material.

Peaks located around 1201 and 1159 cm^–1^ may be related to the asymmetric and symmetric C–O–C stretching vibration of glycosidic linkage from cellulose, respectively,^51,84^ and at 893 cm^–1^, it was possible to identify a peak attributed to the β-(1,4)-glycosidic linkage between glucose units from cellulose chain.^42,50–52,84^ According to the literature, peaks located around 2900 cm^–1^ may be related to the asymmetric stretching of the C–H group present in cellulose and lignin structures.^42,50,52,84^ It was possible to notice that the peak located in this region, in the case of MCC, was divided into two peaks, at 2917 and 2848 cm^–1^, after the HIUS stage, this behavior was also observed by Yuan et al.^50^ Debiagi et al.^42^ suggested that peaks near 1639 and 1600 cm^–1^ can be also associated with the C=C stretching of the aromatic ring in the lignin, which may explain its intensity after the HIUS, and confirms the presence of lignin nanoparticles indicated by the AFM. Regarding the peak near 1455 cm^–1^ may be related to the aromatic skeletal vibration with C–H plane deformation of the aromatic ring present on the lignin structure.^47,51,84^ The presence of a peak at 1246 cm^–1^ indicated a C–O stretching present in the lignin structure.^50^ It was possible to observe a decrease in the bands related to the presence of lignin, which may be due to the disruption of lignin particles by the HIUS mechanism.

As indicated by Guancha-Chalapud et al.,^85^ peaks located near 1430 and 894 cm^–1^ may be related to the presence of crystalline structures. A decrease of these peaks was observed after the HIUS stage, which confirms the indication through XRD analysis by the alteration on crystallinity index. Following Koutsianitis et al.,^77^ the peaks located around 4000–2995, 2900, 1430, 1375, and 900 cm^–1^ are related to physical and chemical alterations on cellulose, such as its crystallinity. They indicated that the ultrasound procedure plays an important role in these properties, where its mechanism enhanced the mass transfer and facilitated the easy penetration of formed radicals into the CMF structure.^77^ Then, it may result in changes of cellulose structure, resulting in a decrease or increase of its crystallinity index, depending on the raw material, as well as other factors that will be discussed in the following sections. The same authors also indicated that the absorbance at 1430 and 894 cm^–1^ are easily affected by the amount of crystalline and amorphous structure of cellulose. Changes in these peaks were observed, aligning with the findings indicated by XRD analysis. The peak located near 1430 cm^–1^ was attributed to the presence of a mixture of crystallized cellulose I and amorphous cellulose. The thermal degradation behavior of MFC and NFC was investigated by TGA and derivative curves (DrTGA), illustrated by Figure 5c,d, respectively. It was noticed that thermal degradation of both samples occurred in three steps (Table 3).

**Table 3:** Thermal Degradation Stages of MFC and NFC

	stage I		stage II		stage III		
	*T* (°C)	mass loss (%)	*T* (°C)	mass loss (%)	*T* (°C)	mass loss (%)	residual mass (%)
CMF	100	5.1	364	66.0	485	23.5	5.4
CNF	100	11.3	280	36.6	485	21.7	30.4

The intensified mass loss at low temperatures observed for CNF can be attributed to increased moisture retention and the presence of hydrophilic residual components, such as small amounts of lignin and low-molecular-weight hemicellulose fragments that were not fully removed during pretreatment.^42^ These constituents typically undergo initial volatilization and degradation near 100 °C, a behavior widely reported for lignocellulosic materials.^21,42,86,87^ The second step, which presents a pronounced mass loss between 250 and 370 °C, suggests reactions of depolymerization, dehydration, and decomposition of glycoside units, which are related to the cellulose decomposition.^42^ A reduction in the cellulose degradation temperature after the HIUS step, from 343.6 to 275.4 °C, was observed, which was expected as a result of reduction in crystallinity index^21,44,87^ previously indicated by the XRD analysis, and, also, suggests a decrease of cellulose chain degree of polymerization due to its break by HIUS mechanism.^18^

Furthermore, the higher final residue after heating to 485 °C indicates the presence of residual lignin or the formation of carbonaceous char. Due to its highly aromatic and recalcitrant structure, lignin decomposes over a broad temperature range and often yields significant fixed-carbon residue, contributing to increased ash and char formation. These findings are consistent with the residual lignin identified by FTIR and AFM, as well as with the enhanced susceptibility of ultrasonicated CNF to carbonization, resulting from its increased surface area. In addition to thermal stability, the drying method also plays a significant role in determining the char content, as fiber rearrangements occur during water removal.^88^ An increase in its intensity is observed after the HIUS procedure, which is observed by other authors. Li et al.,^82^ for example, reported an increase of this parameter from 6.2 to 16.1% after the ultrasonication of commercial cellulose. Supian et al.^89^ detected an elevation in char content from 1.39 to 31.19% after the production of CNF by grinding, where, according to the authors, this change was attributed to the presence of impurities or contamination on the fiber. Then, the results obtained are in accordance with those reported in the literature and indicate the promising potential of black wattle bark residues as a raw material for CNF.

Based on the combined structural results, including the fibrillar morphology observed by AFM, the reduction in crystallinity, and the thermal stability profile, it is possible to outline potential application domains for the produced CNF. The combination of high surface area, nanoscale dimensions, and a greater proportion of amorphous regions may enhance interactions with polymeric matrices, which are advantageous for biodegradable films, hydrogels, and aqueous-based composites. Additionally, the presence of residual lignin, together with the observed thermal behavior, may contribute to barrier performance and increased resistance to char formation, characteristics relevant for coatings, adsorbent materials, and functional systems requiring moderate thermal stability and accessible chemical surfaces. Although further studies are required to validate these possibilities, the results indicate that the CNF obtained here exhibits a set of properties compatible with the technically plausible applications in sustainable materials.

## 4 Conclusions

The results demonstrated that agro-industrial residues of black wattle bark present strong potential as a sustainable source for CNF obtained through an eco-friendly HIUS methodology. Ultrasonication of a 1 wt % cellulose suspension, without a cooling bath, for 30 min enabled effective fibrillation, yielding nanofibrils with diameters 11.93 ± 4.83 nm. The identification of characteristic cellulose functional groups, along with traces of insoluble lignin, confirms the partial removal of noncellulosic components while preserving the nanofibrillar structure. The reduction in crystallinity index from 88.14 to 52.21% highlights the capacity of HIUS-induced cavitation to disrupt ordered domains, whereas thermogravimetric analysis revealed thermal stability changes associated with these structural and morphological modifications.

Beyond confirming the technical feasibility of producing nanofibrils from this recalcitrant biomass, the findings contribute to a broader perspective on the valorization of tannin-rich industrial byproducts and reinforce the potential of HIUS as a green processing route for challenging lignocellulosic residues. The CNF obtained here may serve as promising building blocks for sustainable materials, including biodegradable films, polymeric composites, and food-packaging systems, supporting the development of environmentally responsible alternatives.

Nevertheless, important limitations remain, such as the relatively low overall yield, the persistence of residual lignin, and the processing time required for effective cavitation. These constraints indicate that the HIUS method, although effective, is not fully optimized for maximum efficiency. Future work should explore the combination of HIUS with chemical or enzymatic pretreatments to enhance fibrillation, assess the scalability of the process, and refine mechanical parameters to improve energy efficiency and nanofibril quality. Additionally, evaluating the performance of these CNF in advanced materials will be essential to establishing their applicability in real-world sustainable technologies.
